# Relationship Between Preoperative Surgical Fear, Anxiety, and Satisfaction Levels in Individuals Choosing Bariatric Surgery Tourism: A Descriptive, Cross-Sectional Study

**DOI:** 10.1007/s11695-025-07749-0

**Published:** 2025-03-07

**Authors:** Elif Nazlı Anar, İnci Kırtıl

**Affiliations:** Present Address: Yeditepe University, Istanbul, Turkey

**Keywords:** Bariatric surgical tourism, Surgical fear, Anxiety, Satisfaction, Surgical nursing

## Abstract

**Background:**

Bariatric surgical tourism is a rapidly growing sector. The aim of this study was to evaluate the preoperative surgical fear, anxiety, and satisfaction levels regarding bariatric surgery tourism processes and to examine the relationships between these variables.

**Methods:**

This descriptive, cross-sectional study was conducted between July and November 2024 with foreign patients who visited the general surgery clinic of a healthcare institution for bariatric surgery within the scope of health tourism. Data were collected through face-to-face interviews using the Patient Information Form, Surgical Fear Scale, and Surgical Anxiety Scale. All results were evaluated within a 95% confidence interval, with statistical significance set at *p* < 0.05.

**Results:**

The mean age of the patients was 39.39 ± 9.35 years, and 88.5% were from the UK. Additionally, 88.5% reported choosing bariatric surgery tourism due to high surgical costs in their home country. The mean score for surgical fear was 37.68 ± 20.58, while the mean score for surgical anxiety was 19.53 ± 12.90. Patients who lacked prior knowledge about bariatric surgery tourism had significantly higher surgical fear and anxiety scores (*p* < 0.05). Age, fear and anxiety associated with undergoing surgery in a foreign country, and total surgical anxiety scale score were identified as independent predictors of patients’ satisfaction with their bariatric surgery tourism experience (*R*^2^ = 0.130; *p* < 0.01).

**Conclusions:**

Preoperative knowledge levels and satisfaction with the information provided significantly impacted surgical fear and anxiety levels among bariatric surgery tourism patients.

## Introduction

Obesity is an increasingly significant public health issue worldwide [1]. Weight loss surgery, also known as bariatric surgery, refers to various types of procedures performed to achieve weight loss through systematic and permanent alterations in the digestive system [2].

Medical tourism is defined as the voluntary travel of individuals to another country for medical or surgical treatments. It is a rapidly growing sector driven by the globalization of healthcare services, the pursuit of cost-effective, high-quality medical treatments, and a significant global increase in travel rates [3–5]. Differences in funding allocated for bariatric surgery between countries, waiting times for surgery, lack of insurance coverage, and variations in the application of eligibility criteria limit patients’ access to these procedures [2, 4, 6, 7]. Globally, bariatric surgery tourism is estimated to account for at least 2% of all medical tourism [6, 8]. While multiple factors influence the preference for bariatric surgery tourism, patients also report longer hospital stays compared to their home countries and the availability of 24-h personalized nursing care during their hospitalization as significant reasons for their choice [7].

The incidence of preoperative fear and anxiety varies across different surgical populations, and each patient’s experience of fear and anxiety can be influenced by numerous factors, differing significantly from one individual to another [9]. Preoperative fear and anxiety can affect postoperative wound healing, pain intensity, the required dosage of anesthetic agents, and the need for analgesic medications. Additionally, these negative emotional states may exacerbate the patient's stress response during the postoperative period, potentially leading to various complications or undesirable outcomes [10, 11]. Furthermore, preoperative fear and anxiety significantly impact patient satisfaction throughout the perioperative process [10]. For these reasons, it is essential to incorporate fear and anxiety factors into surgical planning during the preoperative period and ensure that surgical nurses identify and address these emotional factors [10, 12]. Given the widespread prevalence of bariatric surgery tourism in our country, it is crucial to identify the factors influencing anxiety and fear among patients choosing health tourism for bariatric surgery, develop effective care interventions, and implement strategies to enhance patient satisfaction.

However, a review of the existing literature reveals a lack of studies specifically examining preoperative fear, anxiety, and satisfaction levels among patients undergoing bariatric surgery within the context of health tourism, as well as the relationships between these factors. Therefore, this study was designed and conducted to evaluate preoperative surgical fear, anxiety, and satisfaction levels among individuals choosing our country for bariatric surgery tourism and to examine the relationships between these variables.

## Methods

### Study Design

The study was designed and conducted using a descriptive, cross-sectional, and correlational research design.

### Setting

The study was carried out between July and November 2024 in the general surgery clinic of a private healthcare institution in Istanbul, involving international patients undergoing bariatric surgery within the context of medical tourism.

### Sampling

The study population consisted of all foreign patients who underwent bariatric surgery in the designated general surgery clinic (N = 215). The study sample included patients who agreed to participate and met the inclusion criteria. To determine the sample size, data from the institution regarding the number of surgical procedures performed during a similar period in the previous year were used. A known population sample size calculation was performed using an online sample size calculator [13]. Assuming a 95% confidence level and a 5% margin of error, the minimum required sample size was determined to be 139 patients. The study was completed with 156 patients using a non-probability sampling method.

### Inclusion and Exclusion Criteria

Inclusion criteria were as follows: aged 18 years or older, provided informed consent, no diagnosed cognitive and/or psychiatric disorders, no communication barriers, undergoing planned surgical intervention, native English speakers, classified as ASA (American Society of Anesthesiologists) score III or below, and undergoing bariatric surgery for the first time. Patients who did not meet these criteria were excluded from the study.

### Data Collection Tools

Data were collected using a Patient Information Form prepared by the researchers based on relevant literature, along with the Surgical Fear Questionnaire to assess patients’ levels of surgical fear and the Surgical Anxiety Scale to measure preoperative anxiety levels.

#### Patient Information Form

This form, developed by the researchers based on relevant literature [5, 14, 15], consists of two sections. The first section includes questions designed to identify patients’ demographic characteristics. The second section contains questions aimed at assessing patients’ surgical experience, satisfaction levels, and their knowledge and experiences related to bariatric surgical tourism. A numeric rating scale was used to evaluate bariatric surgery patients' satisfaction with surgical tourism, anxiety levels related to surgical tourism, fear levels associated with undergoing surgery in a foreign country, and anxiety levels related to undergoing surgery in a foreign country. Patients were asked to rate their relevant experiences and feelings on a scale from 0 to 10, where 0 represented no experience at all and 10 represented the highest intensity possible. Before completing the form, patients were informed about the meaning of the numerical scale and how to provide their ratings. To ensure the content validity of the form, expert opinions were obtained from four general surgeons specializing in bariatric surgery and four nursing academics with research expertise in bariatric surgery and health tourism.

#### Surgical Fear Questionnaire

This questionnaire was developed by Theunissen et al. (2014) to measure the fear levels caused by surgical interventions among patients undergoing elective surgery. The scale consists of 8 items, rated on an 11-point Likert scale ranging from 0 to 10. Each item is scored as 0 (“not afraid at all”) to 10 (“very afraid”). The scale includes two subdimensions, each containing four items, focusing on short-term fears and long-term fears. The total score for each subdimension is calculated by summing the scores of the respective four items, and the overall scale score is obtained by adding the two subdimension scores. The total score ranges from 0 to 80, with higher scores indicating higher levels of surgical fear. The internal consistency Cronbach's α coefficient of the original scale was reported as 0.89 for the total scale, 0.86 for the short-term outcomes subdimension, and 0.87 for the long-term outcomes subdimension [16]. In this study, the original English version of the scale was used. Cronbach’s α coefficients for reliability in this study were 0.937 for the total scale, 0.926 for the long-term fear subdimension, and 0.918 for the short-term fear subdimension.

#### Surgical Anxiety Scale

This scale was developed by Burton et al. (2019) to assess anxiety levels caused by surgical interventions among patients undergoing elective surgery. It consists of 17 items rated on a 5-point Likert scale ranging from 0 to 4. During the preoperative period, patients rate each item based on how well it reflects their own feelings. The scale comprises three subdimensions: health-related anxiety, recovery-related anxiety, and procedure-related anxiety. Subdimension scores are calculated by summing the respective item scores, along with three additional items not included in any subdimension, to obtain the total scale score. The total score ranges from 0 to 68, with higher scores indicating higher levels of surgical anxiety. The scale does not have a cutoff point. In the original study, the Cronbach’s α coefficient was 0.91 for the total scale, 0.87 for health-related anxiety, 0.78 for recovery-related anxiety, and 0.75 for procedure-related anxiety [17]. In this study, the original English version of the scale was used. Cronbach’s α coefficients for reliability in this study were 0.930 for the total scale, 0.912 for health-related anxiety, 0.923 for recovery-related anxiety, and 0.917 for procedure-related anxiety.

### Data Collection

The study commenced after obtaining all necessary approvals (ethics committee approval, institutional permission, and verbal and written informed consent from participants). Before initiating the data collection phase, a pilot test was conducted with five patients using the forms and scales included in the study to evaluate their feasibility and clarity. Data from the pilot test participants were excluded from the final sample and statistical analysis. Study data were collected by the researcher through face-to-face interviews with international patients undergoing planned bariatric surgical procedures in a clinical setting as part of health tourism. Questions related to demographic characteristics and satisfaction levels from the Patient Information Form, preoperative fear levels from the Surgical Fear Scale, and preoperative anxiety levels from the Surgical Anxiety Scale were administered to patients face-to-face in their hospital rooms upon arrival at the bariatric surgery clinic for their planned surgeries. In addition to the researcher’s proficiency in English speaking and comprehension, a hospital translator accompanied the researcher during data collection. Completing all question forms took approximately 8–10 min per patient. The data collection phase was carefully planned and conducted to avoid disrupting patients’ treatment and care processes as well as the workflow of the surgical clinic.

### Data Analysis

The data collected in the study were analyzed using IBM SPSS Statistics for Windows, Version 22.0 (SPSS INC., Chicago, IL, USA). Frequency and percentage analyses were used to determine the descriptive characteristics of the participants, while mean and standard deviation statistics were employed to examine the scales. The Kolmogorov–Smirnov test was performed to assess the normality of data distribution, and skewness and kurtosis values were also examined. Data were considered normally distributed if these values were within the range of + 2 to − 2 [18, 19]. After confirming normal distribution, parametric statistical methods were used for data analysis. Cronbach’s α coefficients were calculated to determine the reliability of the scales used in the study. Pearson correlation analysis was conducted to determine the relationship between continuous variables. For comparisons of normally distributed continuous variables between two groups, the independent samples t-test was used, while one-way ANOVA was employed for comparisons between three or more groups. In cases where ANOVA revealed significant differences, Tukey and LSD post hoc analyses were performed to identify the source of these differences. Multiple linear regression analysis was used to assess the effects of independent variables on dependent variables. Multicollinearity among independent variables was checked using Variance Inflation Factors (VIF) and tolerance values, with VIF values below 10 and tolerance values above 0.1 considered acceptable. Autocorrelation among error terms was assessed using the Durbin-Watson statistic, with values between 1.5 and 2.5 considered acceptable [20]. Before conducting multiple linear regression analyses, assumptions of normality, homoscedasticity, and multicollinearity were verified to ensure they were not violated. All results obtained from the study were evaluated at a 95% confidence interval, with statistical significance set at *p* < 0.05.

### Ethical Principles

Before starting the study, ethical approval was obtained from Non-Interventional Clinical Research Ethics Committee of Yeditepe University (dated 28.06.2024, with approval number E.83321821-805.02.03-432) and institutional permission from the organization where the study would be conducted (dated 22.07.2024, with approval number 694/2024). All patients who volunteered to participate in the study were thoroughly informed about the research, and verbal and written informed consent was obtained. Written permissions for the scales used in the study were also obtained from the authors who developed them via email.

## Results

In the study, the mean age of the patients was determined to be 39.39 ± 9.35 years (range, 18–64). The majority of patients (88.5%) were from the UK, followed by 4.5% from Portugal, 3.2% from Malta, and 0.6% from the Netherlands. While 25.6% of the patients reported having a chronic illness, 74.4% stated they had no chronic health conditions. Diabetes was identified as the most common chronic illness, with a prevalence of 30% (Table 1).

**Table 1:** Clinical characteristics of the patients, surgical process, and their knowledge and experience regarding bariatric surgical tourism (N = 156)

Variables	*n*	%
Age		
30 and below	28	17.9
31–40	65	41.7
41–50	42	26.9
51 and above	21	13.5
Gender		
Female	133	85.3
Male	23	14.7
Education level		
High school	99	63.5
Bachelor’s degree	43	27.6
Postgraduate degree	14	9.0
Country of residence		
UK	138	88.5
Portugal	7	4.5
Malta	5	3.2
Netherlands	1	0.6
Other	5	3.2
Presence of chronic disease		
Yes	40	25.6
No	116	74.4
Current chronic diseases (*n* = 40)*		
Diabetes	12	30.0
Hypertension	9	22.5
Thyroid	8	20.0
Sleep apnea	4	10.0
Other	15	37.5
Satisfaction with the information about the surgical intervention in the preoperative period		
Undecided	7	4.5
Satisfied	41	26.3
Very satisfied	108	69.2
The importance of nursing care in the surgical process		
Important	5	3.2
Very important	151	96.8
Satisfaction with preoperative nursing care		
Not satisfied at all	1	0.6
Undecided	5	3.2
Satisfied	31	19.9
Very satisfied	119	76.3
Communication with the multidisciplinary health team before surgery		
Yes	130	83.3
No	26	16.7
Communicated team members (*n* = 130)*		
Nurse	112	86.2
Surgeon	99	76.2
Anesthesiologist	86	66.2
Dietitian	40	30.8
Physiotherapist	11	8.5
Thinking that bariatric surgery is a teamwork		
Yes	145	92.9
Undecided	11	7.1
Preoperative knowledge about bariatric surgical tourism		
Yes	83	53.2
No	20	12.8
Partially	53	34.0
Information about the importance of routine postoperative controls		
Yes	124	79.5
No	4	2.6
Partially	28	17.9
Knowledge about postoperative risks and complication management		
Yes	128	82.1
No	4	2.6
Partially	24	15.4
Understanding and satisfaction with the information given about surgical tourism and surgical processes		
Undecided	6	3.8
It was clear and I was satisfied	65	41.7
It was very clear and I was very satisfied	85	54.5
Reasons for choosing to travel abroad for bariatric surgery*		
Higher costs of surgery in the country of residence	138	88.5
Recommendation by previously satisfied people	83	53.2
Waiting time for surgery in the country of residence is too long	61	39.1
Inability to meet the criteria for bariatric surgery in the country of residence	45	28.8
Expect to have a better service	38	24.4
Expect it to be more economical	13	8.3
Refusal of the family to undergo surgery	3	1.9
Fear of stigmatization	3	1.9
Other reasons	9	5.8
Postoperative frequency plan to maintain routine controls in their own country		
Once a month	107	68.6
Every 1–3 months	21	13.5
Every 3–6 months	28	17.9
	**Mean ± SD**	**Min–Max**
Age	39.39 ± 9.35	18–64
Satisfaction levels with surgical tourism	8.90 ± 1.34	3–10
Anxiety level related to surgical tourism	5.86 ± 3.19	0–10
Fear level related to having surgery in a different country	5.62 ± 3.10	0–10
Anxiety level related to having surgery in a different country	5.47 ± 3.03	0–10

*SD*, Standard deviation

^*^ More than one selected item

The majority of patients (69.2%) reported being very satisfied with the information provided to them about the surgical procedure during the preoperative period. Regarding satisfaction with nursing care processes during the preoperative phase, 76.3% of patients stated they were very satisfied. It was found that 83.3% of patients had communicated with at least one member of the surgical team before the surgery, 53.2% had knowledge about bariatric surgical tourism before the procedure, 82.1% were informed about managing potential risks and complications after bariatric surgery, and 79.5% understood the importance of routine follow-up appointments in the postoperative period. Additionally, 54.5% of participants reported that the explanations they received regarding the surgical process were highly comprehensible, and they expressed very high satisfaction with the overall process. When examining the reasons why participants chose to undergo bariatric surgery abroad, 88.5% cited higher surgical costs in their home country, 53.2% mentioned recommendations from previously satisfied individuals, 39.1% highlighted excessively long waiting times for surgery in their home country, and 28.8% noted they did not meet bariatric surgery criteria in their home country. Furthermore, 24.4% believed they would receive better care in Turkey, 8.3% perceived the procedure as more cost-effective in Turkey, 1.9% were concerned about stigma in their home country, and 1.9% reported family opposition to the surgery as reasons for choosing health tourism (Table 1).

The patients’ mean total score on the Surgical Fear Scale was 37.68 ± 20.58 (range: 0–73), while the mean total score on the Surgical Anxiety Scale was 19.53 ± 12.90 (range: 0–56). When examining the relationship between patients’ levels of surgical fear and anxiety, a statistically significant and positive correlation was observed between the total scores and the sub-dimensions of both scales (*p* < 0.01). Detailed results regarding the correlations between the Surgical Fear Scale, Surgical Anxiety Scale, satisfaction levels, and selected variables are presented in Table 2.

**Table 2:** Results of the relationships between the Surgical Fear Scale, Surgical Anxiety Scale and satisfaction scores of the patients and some variables (N = 156)

Variables		Age	Satisfaction levels with surgical tourism	Anxiety level related to surgical tourism	Fear level related to having surgery in a different country	Anxiety level related to having surgery in a different country
Age	*r*	**-**	− 0.179*	− 0.070	− 0.109	− 0.123
	*p*		0.026	0.385	0.177	0.128
Surgical fear scale total	*r*	− 0.119	− 0.156	0.435**	0.728**	0.634**
	*p*	0.139	0.052	0.000	0.000	0.000
Long term fear	*r*	− 0.180*	− 0.085	0.409**	0.727**	0.645**
	*p*	0.025	0.294	0.000	0.000	0.000
Short term fear	*r*	− 0.050	− 0.199*	0.403**	0.635**	0.544**
	*p*	0.532	0.013	0.000	0.000	0.000
Surgical anxiety scale total	*r*	− 0.083	− 0.218**	0.292**	0.592**	0.534**
	*p*	0.302	0.006	0.000	0.000	0.000
Concerns about health	*r*	− 0.082	− 0.174*	0.254**	0.554**	0.486**
	*p*	0.308	0.030	0.001	0.000	0.000
Concerns about recovery	*r*	− 0.020	− 0.260**	0.303**	0.463**	0.422**
	*p*	0.806	0.001	0.000	0.000	0.000
Concerns about procedures	*r*	− 0.112	− 0.171*	0.234**	0.549**	0.516**
	*p*	0.166	0.033	0.003	0.000	0.000
Anxiety level related to surgical tourism	*r*	− 0.070	− 0.110	-	0.405**	0.440**
	*p*	0.385	0.173		0.000	0.000
Fear level related to having surgery in a different country	*r*	-	-	-	-	0.869**
	*p*					0.000

*r*, Correlation coefficient; *p*, Significance value * < 0.05 ** < 0.01 (Pearson correlation analysis)

When comparing the mean total scores of bariatric surgery patients on both the Surgical Fear Scale and the Surgical Anxiety Scale with independent variables in the study, statistically significant results were found only for the variable ‘having knowledge about bariatric surgery tourism before surgery.’ According to the analysis, patients who lacked knowledge about bariatric surgery tourism before the operation had significantly higher mean total scores on both the Surgical Fear Scale and the Surgical Anxiety Scale (*p* < 0.05). Variables such as age, gender, education level, presence of chronic disease, preoperative communication with multidisciplinary healthcare team members, perception of bariatric surgery as a team effort, and plans to continue routine follow-up after returning to their home country did not show a statistically significant difference in the total and sub-dimension scores of the Surgical Fear and Anxiety Scales (*p* > 0.05).

Multiple linear regression analyses were conducted using the variables that were statistically significant in pairwise comparisons to identify the factors affecting the mean scores of patients’ Surgical Fear Scale, Surgical Anxiety Scale, and satisfaction levels with their bariatric surgery tourism experience. It was found that anxiety levels related to surgical tourism and fear levels associated with undergoing surgery in a foreign country were significant independent predictors of patients’ fear levels (*R*^2^ = 0.549; *F* = 38.774; *p* < 0.001) (Table 3). Additionally, satisfaction levels with surgical tourism and fear levels associated with undergoing surgery in a foreign country were identified as significant predictors of patients’ preoperative anxiety levels (*R*^2^ = 0.394; *F* = 17.787; *p* < 0.001) (Table 4). Age, fear levels associated with undergoing surgery in a foreign country, anxiety levels associated with undergoing surgery in a foreign country, and total surgical anxiety scores were also determined to be independent variables significantly influencing patients’ satisfaction levels with their bariatric surgery tourism experience (*R*^2^ = 0.130; *F* = 3.169; *p* < 0.01) (Table 5).

**Table 3:** Factors affecting the mean scores of the patients on the Surgical Fear Scale

Independent variables	Unstandardized coefficients		Standard coefficients	*t*	*p*	95% Confidence interval	
	B	SE	β				
Constant	12.896	4.275		3.016	0.003	4.448	21.343
Knowledge on bariatric surgical tourism**	− 6.128	3.557	− 0.149	− 1.723	0.087	− 13.155	0.000
Anxiety level related to surgical tourism	1.218	0.395	0.189	3.079	0.002*	0.436	1.999
Fear level related to having surgery in a different country	4.614	0.733	0.696	6.293	0.000*	3.165	6.062
Anxiety level related to having surgery in a different country	− 0.517	0.768	− 0.076	− 0.674	0.502	− 2.034	1.000

*R* = 0.751; *R*^2^ = 0.549; *F* = 38.774; *p* = 0.000; Durbin-Watson = 2.110

^**^Dummy variable (Reference = No), *SE*, Standard error, *p*, Significance, *p < 0.05

**Table 4:** Factors affecting the mean scores of the patients on the Surgical Anxiety Scale

Independent variables	Unstandardized coefficients		Standard coefficients	*t*	*p*	95% Confidence interval	
	B	SE	β				
Constant	26.961	6.517		4.137	0.000	14.084	39.839
Knowledge on bariatric surgical tourism**	− 4.989	2.585	− 0.194	− 1.930	0.055	− 10.096	0.118
Satisfaction levels with surgical tourism	− 1.787	0.615	− 0.187	− 2.905	0.004*	− 3.003	− 0.572
Anxiety level related to surgical tourism	0.342	0.288	0.085	1.190	0.236	− 0.226	0.910
Fear level related to having surgery in a different country	2.367	0.536	0.570	4.414	0.000*	1.308	3.427
Anxiety level related to having surgery in a different country	− 0.256	0.565	− 0.060	− 0.453	0.651	− 1.373	0.861
*R* = 0.646; *R*^2^ = 0.394; F = 17.787; *p* = 0.000; Durbin-Watson = 2.073							

^**^Dummy variable (Reference = No), *SE*, Standard error, *p*, Significance, *p < 0.05

**Table 5:** Factors affecting patients’ satisfaction with bariatric surgical tourism experience

Independent variables	Unstandardized coefficients		Standard coefficients	*t*	*p*	95% Confidence interval	
	B	SE	β				
Constant	10.399	0.552		18.847	0.000	9.309	11.489
Age	− 0.030	0.011	− 0.207	− 2.671	0.008*	− 0.052	− 0.008
Knowledge on bariatric surgical tourism**	0.354	0.212	0.131	1.668	0.097	− 0.065	0.772
Anxiety level related to surgical tourism	− 0.016	0.037	− 0.037	− 0.416	0.678	− 0.089	0.058
Fear level related to having surgery in a different country	0.162	0.077	0.374	2.122	0.035*	0.011	0.314
Anxiety level related to having surgery in a different country	− 0.142	0.071	− 0.319	− 1.996	0.048*	− 0.282	− 0.001
Surgical fear scale total score	− 0.001	0.010	− 0.021	− 0.142	0.887	− 0.020	0.018
Surgical anxiety scale total score	− 0.026	0.013	− 0.248	− 2.028	0.044*	− 0.051	− 0.001

*R* = 0.361; *R*^2^ = 0.130; *F* = 3.169; *p* = 0.004; Durbin-Watson = 2.041

^**^Dummy variable (Reference = No), *SE*, Standard error, *p*, Significance, *p < 0.05

## Discussion

The literature indicates that countries such as Mexico, Romania, and Turkey have emerged as leading bariatric tourism destinations, particularly attracting patients from the USA, the UK, and Germany [6, 21]. An international study also reported that individuals choosing Turkey for bariatric surgical tourism predominantly come from Germany, the USA, Cyprus, Nigeria, Saudi Arabia, Iraq, the UK, Sweden, and Canada [6]. In another study on the topic, an analysis of survey responses from the top five countries revealed that Turkish surgeons primarily treat German and British bariatric surgery patients [22]. In the present study, it was determined that the majority of patients, based on their country of residence, came from the UK. These findings align with the literature, confirming that patients from high-income countries, such as the UK, often prefer destinations where costs are lower or waiting times are shorter. Factors contributing to Turkey’s appeal include cultural compatibility, language support, the increasing number of medical tourism agencies, its proximity to European countries, and the quality of treatment and care services. Additionally, the exchange rate plays a significant role in country selection, substantially influencing costs from the patient’s perspective. In conclusion, cross-border patient mobility in bariatric surgery tourism is shaped by economic, geographical, cultural, and healthcare quality-related factors.

Globally, patients may choose bariatric surgery tourism for a variety of reasons. Differences in financial coverage allocated to bariatric procedures between countries, waiting times for surgery, lack of insurance coverage for such procedures, or variations in eligibility criteria can limit patients’ access to these surgeries [2, 4, 6, 7]. Studies report that treatment costs in developing countries can be approximately 20–30% of those in the USA [7]. One study highlighted that publicly funded bariatric surgical interventions account for fewer than 1 per 100,000 people annually and that these services meet far less than 0.1% of the actual need [23]. In countries like Canada, factors such as average waiting times of 2–3 years between referral for bariatric surgery and the scheduled surgery date, or the perception that patients do not meet surgical criteria, have been reported as drivers for patients seeking bariatric surgery tourism [2, 7]. In countries with national health programs, such as Canada and the UK, insurance coverage limitations are also highlighted as significant factors contributing to bariatric surgery tourism [7]. A case study reported that a patient opted for bariatric surgery tourism due to lower costs and minimal approval processes compared to their home country [8]. Another study identified family or friends' opinions or recommendations as the most influential factor in patients' decision-making process regarding health tourism [24]. Consistent with the literature, this study found that while bariatric surgery patients cited various reasons for engaging in health tourism, the leading factors included higher surgical costs in their home countries, recommendations from previously satisfied individuals, prolonged waiting times for surgery, and failure to meet the eligibility criteria for bariatric surgery in their own countries. These findings indicate that Turkey offers shorter waiting times, cost-effective treatment and care options, and has consequently become a highly recommended destination for health tourists.

Surgical tourism patients may experience fear and anxiety during the process due to factors such as travel anxiety, uncertainty, insecurity, language barriers, negative prejudices about the destination country and institutions, and mismatches between expectations and facilities, services, and costs [25–27]. It has been reported that fear or anxiety is one of the primary factors discouraging many patients from traveling abroad, with the lack or inadequacy of information sources identified as the main reason for these emotions. Additionally, distance can also serve as a source of concern and fear for health tourists, leading patients to prefer destinations in closer proximity solely for this reason [24]. Studies have identified language barriers as one of the most significant obstacles for health tourists, noting that these barriers can lead to misunderstandings between patients and healthcare professionals, subsequently causing anxiety among patients [28]. Negative emotions such as preoperative fear and anxiety can also affect patient satisfaction throughout the perioperative experience [10]. At the same time, patient satisfaction with medical tourism serves as an important indicator of healthcare service quality and predicts patients' intention to revisit. Factors such as the responsibility and attitude of healthcare providers, perceived value, and additional elements like environment, food, and communication significantly shape health tourists’ perceptions of healthcare quality, thereby influencing their overall experience and satisfaction [28]. For these reasons, planning the surgical process in the preoperative period while considering fear and anxiety related to surgical intervention is crucial for enhancing patient satisfaction [10, 12]. Furthermore, feelings of fear and anxiety are often intertwined in surgical patients. Studies conducted with surgical patients have shown that individuals experiencing preoperative fear exhibit significant differences in their anxiety scores [29, 30]. As a result of this study, it was determined that bariatric surgery patients reported a high level of satisfaction with surgical tourism, while their fear and anxiety levels related to surgical tourism were moderate. Statistically significant relationships were found between patients’ levels of fear, anxiety, and satisfaction. The discussion remains limited because studies conducted with individuals opting for bariatric surgical tourism are scarce, and factors such as fear and anxiety have not been adequately addressed in this patient population. Nevertheless, considering the impact of patients’ fear and anxiety on their satisfaction levels, the present study highlights the importance of effectively managing fear and anxiety through a multidisciplinary approach in the preoperative period for health tourists. In particular, the attitude of healthcare providers and strengthening communication between patients and service providers appear to be critical for improving the healthcare tourism experience.

Surgical procedures can cause varying levels of anxiety in patients due to concerns such as fear of death, not waking up from anesthesia, and postoperative discomfort [30, 31]. In a qualitative study conducted with bariatric surgery tourism patients, participants expressed feelings of anxiety and disappointment during the preoperative period due to insufficient information about the surgery and the requirements of medical tourism [15]. It has been reported that 25.64% of bariatric patients traveling for surgery exhibited anxiety symptoms preoperatively, while 40% experienced such symptoms postoperatively [32]. In studies with bariatric surgical patients, it has been reported that patients experience severe anxiety in the preoperative period for different reasons [33, 34]. In the present study, although bariatric surgery tourism patients exhibited low-to-moderate levels of surgical anxiety, those who lacked preoperative knowledge about bariatric surgery tourism had significantly higher anxiety levels. Independent predictive factors affecting surgical anxiety levels among bariatric surgery patients were identified as fear associated with undergoing surgery in a foreign country and satisfaction with surgical tourism. These findings suggest that preoperative education and psychological support can help reduce surgical anxiety levels in bariatric surgery tourism patients, thereby enhancing overall patient satisfaction.

Patient satisfaction holds significant importance for both surgical outcomes and postoperative recovery [5]. The literature emphasizes that healthcare institutions offering bariatric medical tourism services in the destination country must deliver better outcomes and higher patient satisfaction compared to the patients’ home countries; otherwise, patient results and experiences may be negatively impacted [22]. Another study focusing on healthcare tourism patients highlighted that the quality of healthcare services plays a crucial role in patient satisfaction and quality of life [35]. Patient satisfaction is considered a key indicator reflecting both individual experiences and the success of healthcare providers in bariatric surgery tourism. Therefore, it is essential for service providers to develop communication-focused approaches aimed at enhancing patient satisfaction, overcoming language barriers, and offering personalized support [24, 28]. A study examining the satisfaction of healthcare tourism patients in the Czech Republic revealed that most tourists expressed satisfaction with the services provided. The primary reasons for satisfaction included a clear and attentive approach, hygienic environments, low costs, and high-quality medical care. However, approximately one-third of patients reported dissatisfaction due to communication gaps and perceived deficiencies in service quality. Patients specifically emphasized the need for easier access to information regarding postoperative care and processes, highlighting that a communication-focused approach could enhance patient satisfaction [24]. In contrast, the satisfaction rate among patients traveling to Pakistan for bariatric surgery tourism was reported as 92.3%; however, the factors associated with satisfaction were not examined in that study [5]. A systematic review examining patient experiences in medical tourism highlighted that healthcare service quality, the quality of interactions with service providers, the positive attitudes of healthcare professionals, the cleanliness of physical environments, effective overcoming of language barriers, and receiving high-quality care despite low costs significantly enhance the experiences and satisfaction of health tourists [28]. In the literature, no study specifically examining the impact of fear and anxiety levels on satisfaction among bariatric surgery tourism patients has been identified. Addressing this gap, the present study determined that most patients were generally satisfied with their bariatric surgery tourism experience, reporting predominantly positive outcomes. The most significant independent predictive variables affecting patient satisfaction were identified as age, total score on the surgical anxiety scale, and fear and anxiety levels associated with undergoing surgery in a foreign country. These findings align with previous studies, emphasizing that patient satisfaction is influenced not only by medical outcomes but also by psychosocial and sociodemographic factors.

### Limitations

This study was conducted exclusively with patients undergoing bariatric surgery at a single private healthcare institution; therefore, the findings can only be generalized to this specific population. Additionally, the reliance on participants' self-reported data and the lack of in-depth investigation into the reasons behind patient satisfaction are considered limitations of the study. One of the limitations of the study is that patients' past experiences of surgical tourism were not questioned, because experiences may also affect the levels of fear and anxiety in this area.

## Conclusion

In this study, conducted to evaluate the levels of preoperative surgical fear, anxiety, and satisfaction with bariatric medical tourism processes among individuals who chose Turkey for bariatric surgery within the scope of medical tourism, as well as to examine the relationships between these variables, it was observed that the vast majority of bariatric medical tourism patients had a high level of knowledge in the preoperative period. Participants reported being highly satisfied with the information provided and stated that they had received detailed explanations regarding bariatric surgery tourism, the importance of routine postoperative follow-ups, postoperative risks, and complication management. The study found that patient satisfaction levels with bariatric surgery tourism were notably high, while fear and anxiety levels related to surgical tourism were moderate. Statistically significant relationships were identified between fear, anxiety, and satisfaction levels. Additionally, patients who had sufficient knowledge about bariatric surgery tourism in the preoperative period demonstrated significantly lower levels of surgical fear and anxiety. The most significant independent predictive variables affecting patient satisfaction were identified as age, the total score on the surgical anxiety scale, and levels of fear and anxiety experienced due to undergoing surgery in a foreign country.

In the context of bariatric medical tourism, these findings highlight the critical role of preoperative education and counseling services in reducing surgical fear and anxiety levels and enhancing patient satisfaction. Specifically, increasing patients’ knowledge about postoperative processes, raising awareness regarding complication management, and emphasizing the importance of routine follow-up care can improve both individual and systemic patient outcomes. Surgical nurses should develop tailored educational programs designed to address the specific needs of bariatric surgery tourism patients, monitor their implementation, and ensure the continuity of these programs. Moreover, to address the fear and anxiety associated with undergoing surgery in a foreign country, nurses should adopt a culturally sensitive and empathetic care approach. Such an approach can enhance patient trust, strengthen the nurse-patient relationship, and improve the overall healthcare tourism experience. Randomized controlled trials could be conducted to evaluate the effects of individualized, culturally tailored educational plans or psychoeducation programs implemented by surgical nurses during the preoperative period on surgical fear, anxiety, and patient satisfaction in bariatric surgical tourism patients. Additionally, interventional studies could be carried out to investigate the impact of surgical team members' communication skills on patient satisfaction, as well as levels of fear and anxiety.

## Data Availability

The data of this study are available from the corresponding author upon reasonable request.
